# Preclinical Evaluation of White Led-Activated Non-porphyrinic Photosensitizer OR141 in 3D Tumor Spheroids and Mouse Skin Lesions

**DOI:** 10.3389/fonc.2018.00393

**Published:** 2018-09-21

**Authors:** Bastien Doix, Estelle Bastien, Alix Rambaud, Adán Pinto, Caroline Louis, Vincent Grégoire, Olivier Riant, Olivier Feron

**Affiliations:** ^1^Pole of Pharmacology and Therapeutics (FATH), Institut de Recherche Expérimentale et Clinique, Université catholique de Louvain, Brussels, Belgium; ^2^Pole of Molecular Imaging, Radiotherapy and Oncology, Institut de Recherche Expérimentale et Clinique, Université catholique de Louvain, Brussels, Belgium; ^3^Institute of Condensed Matter and Nanosciences Molecules, Solids and Reactivity (IMCN/MOST), Université catholique de Louvain, Louvain-la-Neuve, Belgium

**Keywords:** photodynamic therapy, photosensitizer, daylight, skin cancer, actinic keratosis, 3D model, spheroids

## Abstract

Photodynamic therapy (PDT) is used to treat malignancies and precancerous lesions. Near-infrared light delivered by lasers was thought for a while to be the most appropriate option to activate photosensitizers, mostly porphyrins, in the depth of the diseased tissues. More recently, however, several advantages including low cost and reduced adverse effects led to consider light emitting diodes (LED) and even daylight as an alternative to use PDT to treat accessible lesions. In this study we examined the capacity of OR141, a recently identified non-porphyrin photosensitizer (PS), to exert significant cytotoxic effects in various models of skin lesions and tumors upon white light activation. Using different cancer cell lines, we first identified LED lamp as a particularly suited source of light to maximize anti-proliferative effects of OR141. We then documented that OR141 diffusion and light penetration into tumor spheroids both reached thresholds compatible with the induction of cell death deep inside these 3D culture models. We further identified Arlasove as a clinically suitable solvent for OR141 that we documented by using Franz cells to support significant absorption of the PS through human skin. Finally, using topical but also systemic administration, we validated growth inhibitory effects of LED-activated OR141 in mouse skin tumor xenograft and precancerous lesions models. Altogether these results open clinical perspectives for the use of OR141 as an attractive PS to treat superficial skin malignant and non-malignant lesions using affordable LED lamp for photoactivation.

## Introduction

Photodynamic therapy (PDT) has been around for several decades in diverse areas of medicine to treat diseases (1, 2). In oncology, first and second generations of photosensitizers are approved to treat various cancers, in particular skin cancers since easily accessible to light illumination (3). In the non-melanoma skin cancer field, basal cell carcinoma (BCC) is the most responsive to PDT (4) while squamous cell carcinoma (SCC) is rather treated by more direct approaches such as surgery but can be treated by PDT when *in situ* and not too deep and extended nor invasive (5, 6). Non-cancerous lesions like actinic keratosis (AK) which can evolve in aggressive SCC in 10–20% of the cases, also represents attractive targets for PDT (7, 8). Other oncological indications for PDT include hollow organ tumors like oral (9), oesophageal (10), bladder and prostate cancers accessible through endoscopy and a few others that may be exposed to the required illumination at the time of surgery such as mesothelioma (11) or glioblastoma (12, 13).

One of the main theoretical advantage of PDT over other anticancer modalities like chemotherapy (CT) or radiotherapy (RT) is a reduced extent of adverse events (AE). Indeed, since PDT needs both the administration of a PS (in theory not toxic *per se*) and a consecutive targeted light irradiation (rendering the PS toxic at the desired site), cytotoxicity will occur more specifically in tumors rather than within surrounding healthy tissues. This theoretical advantage is however limited to local administration of PS since systemic administration of currently available PS exposes patients to pain and photosensitivity-related side effects (skin and eyes in particular) (14, 15). On the other hand one drawback that is accounting for the limited clinical use of PDT (even to target precancerous lesions) is related to the need of dedicated light sources based most of the time on expensive near infrared (NIR) lasers (16) (often restricted to few clinical centers) in order to work at highly penetrating wavelengths. The above disadvantages of high-irradiance light sources have lately led the field to consider the possibility to use blue and white light delivered by LED lamps and even daylight for PDT (17, 18). This mode of illumination should expand the potential of PDT to treat lesions (in particular those covering large body areas) while reducing pain associated with laser-based photoactivation and thereby increasing patient tolerance (8, 19).

We recently identified a new photosensitizer named OR141 and further unraveled that its effects resulted from the oxidation of various ER-related proteins including mTOR but also of two major proteasomal deubiquitinases, the inactivation of which further exacerbating the ER stress induced by PDT (20). OR141 was further identified to accumulate in the ER and to generate highly reactive singlet oxygen (resulting from type II reactions) in the presence of minimal amounts of O_2_, making it particularly suited to be used in the hypoxic environment of tumors. In addition, the short OR141 half-life in the body offers a good safety profile, dramatically reducing the potential photosensitivity following its administration (20). Still, to prove the clinical potential of this new PS, evidence for an efficient delivery and ease of use with affordable light sources should be provided. In this study, we therefore chose to explore the capacity of OR141 to be used for PDT under day-light or LED conditions in a clinically-proof formulation to treat mouse models of squamous cell carcinoma and actinic keratosis. The current validation of OR141 as a white-light activated PS exhibiting potent cytotoxic effects both *in vitro* and *in vivo* models opens perspectives for a broader use of PDT to treat superficial precancerous and cancer lesions.

## Materiels and methods

### Cell cultures

All tumor cell lines (human skin A431, mouse B16 melanoma and SCC7 squamous cell carcinoma) were initially acquired from collections where they are regularly authenticated by short tandem repeat profiling. Cells were used within 3 months after resuscitation of frozen aliquots and regularly checked for mycoplasma contamination. For PDT, cells were washed and incubated in normal medium with OR141 at desired concentration ranging from 0.01 to 100 μM for 1 h and photoactivated by a day-light LED source (30 W equivalent) for 1 h. In some experiments, a halogen light source (KL5000 LCD) as well as direct sunlight exposure were also used. Cell growth was analyzed in colorimetric assays using PrestoBlue reagent (Invitrogen, Waltham, MA, USA) (21). Briefly, 24 h after treatment, cells were washed with PBS before incubation with PrestoBlue reagent (diluted 10-fold in cell medium) for 60 min. Normalization was performed against untreated cells (i.e., 100% viability) while background signal from wells without cells was fixed as 0%.

### 3D tumor spheroids

Spheroids were prepared with SCC7 or A431 cells by seeding 1,500 cells/well in Ultra-Low Attachment 96-well plate (Corning) in DMEM supplemented with 10% heat-inactivated FBS. Spheroid growth was monitored using live-cell phase contrast microscope (Axio Observer, Zeiss). For PDT, spheroids were incubated in normal medium with OR141 at desired concentration ranging from 0.01 to 100 μM for 4 h and photoactivated by a day-light LED source (30 W equivalent) for 90 min. The effects of LED-photoactivated OR141 on spheroid growth were determined using PrestoBlue reagent as detailed above except that spheroids were incubated for 24 h (6 spheroids per condition). For cell death measurements, spheroids were exposed to a 2 mg/ml propidium iodide (PI) solution for 5 min at room temperature and after two washes with PBS, spheroids were placed in an acquisition dish (CellView glass bottom dish, Greiner). PI staining and OR141 autofluorescence were measured with a confocal fluorescence microscope Cell Observer Spinning Disk (COSD) Zeiss (laser at 561 nm and bandpass filter 617/73 nm and laser at 488 nm and bandpass filter 520/35 nm, respectively); z-stacking was performed on each spheroid to select images at 80 μm of depth and fluorescence signals were analyzed using ImageJ software as previously reported (22). Briefly, images of each spheroid were uploaded in the software and an in-house plugin was run to divide them into 50 concentric rings going from the rim to the center. Fluorescence intensity within each ring was then quantified using Image J dedicated tool (3 spheroids per condition). In some experiments, spheroids were digested and the extent of PI-positive cells was determined using flow cytometry. For this purpose, cells were isolated by trypsin digestion for 5 min at 37°C and after washing in FACS buffer (PBS, 2% BSA, 1 mM EDTA), were stained with 1 μg/ml PI for 15 min before acquisition (10 replicates per condition). LED-exposed untreated spheroids but also OR141-treated spheroids maintained in the dark were used as controls.

### Maximal solubility and skin penetration assays

Maximum solubility of OR141 in solvents was determined by HPLC according to a method previously reported (23). Franz's cells (Citoxlab) were used to determine the penetration of a 1 mg/ml solution of OR141 through human skin (1 μg per cm^2^); the amount that traversed the skin was retrieved in the receptor fluid (Arlasolve) and the amount that stayed in the epiderma and the derma was extracted for quantification as above (20, 23).

### *In vivo* experiments

All the experiments involving mice received the approval of the University Ethic Committee (approval ID 2016/UCL/MD018), and were carried out according to National Animal Care regulations. All mice were obtained from Elevage Janvier, LeGenest-St-Isle, France. Tumor xenografts were initiated by injecting subcutaneously 1 ^*^ 10^6^ A431 cells in the flank of 7-week-old nude NMRI mice (*n* = 5 per condition). Tumors were allowed to grow until 20 mm3 before OR141 treatment. For the carcinogenesis model, a two-step, well-described (24), chemical induction protocol using DMBA (7,12-dimethylbenz[a]anthracene) and TPA (12-O-tetradecanoylphorbol-13-acetate) was used. Briefly 3-week-old FVB mice were anesthetized and their back shaved. For initiation, 50 μg of DMBA diluted in acetone were applied on day 0, 2, and 4. A 10-days resting period was observed before proceeding with the promotion phase using 4 μg of TPA two times a week for 10 weeks; at this stage, all the mice developed 10–15 small papilloma-type lesions on their back. For treatments, OR141 was either administered intraperitoneally (40 mg/kg in Solutol/DMSO/NaCl 0.9%) or topically applied (200 μl of a 10 mg/ml solution in Super Refined® Arlasolve, Croda), 4 h and 30 min, respectively, before a 30-min tumor illumination with a 30 W equivalent day-light LED.

### Statistical analysis

Data are expressed as mean ± s.e.m.; a minimum of three experiments were carried out for each experimental condition. The statistical significance between treatments was determined by Student's *t*-test when comparing two groups and one-way analysis of variance (ANOVA, Bonferroni's *post hoc* test) when comparing multiple groups. All data were analyzed with the GraphPad Prism 7.0 software (San Diego, CA, USA).

## Results

### LED-activated OR141 induces cancer cell death

**Figure 1:**
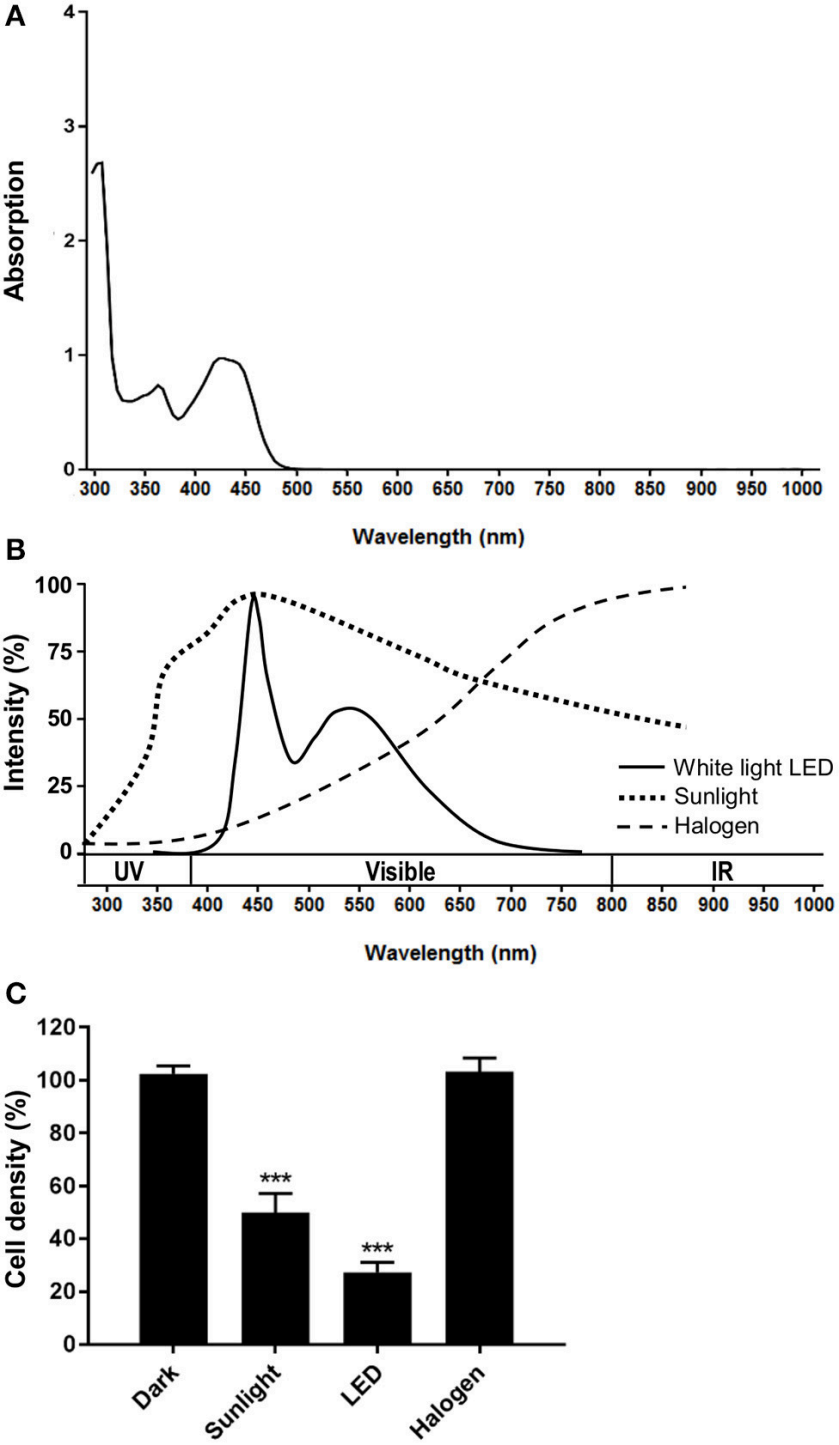
Optimal OR141 photoactivation by white LED illumination. **(A)** OR141 absorption spectrum. **(B)** Emission spectra of different light sources: sunlight, cool white light LED and halogen. **(C)** Growth inhibitory effects of 1 μM OR141 upon activation by exposing A431 skin cancer cells to the indicated light sources. ***P < 0.001, *n* = 3.

We first determined the absorption spectrum of OR141 and identified two peaks including one in the visible light range at 450 nm (Figure 1A). As shown in Figure 1B, this profile actually fits the emission spectrum of a LED light to a larger extent than that of a halogen light (max within red and infrared wavelengths). When comparing these different sources of illumination, LED light exposure actually led to a more pronounced reduction in the viability of OR141-treated human epidermoid A431 cancer cells than the other light sources (Figure 1C and Table 1). We next examined the dose-dependent cytotoxic effects of LED-activated OR141 in A431 cancer cells as well as in two other cancer cell types, namely melanoma B16 cells and squamous cell carcinoma cells SCC7. In these different cell lines, we found EC50 values in the μM range upon OR141 photoactivation (as determined 24 h after 1 h light illumination) (Table 1). By contrast, a lack of cytotoxicity even at 100 μM OR141 prevented the calculation of EC50 when OR141-treated cancer cells were maintained in the dark (Table 1).

**Table 1:** EC50 values related to the growth inhibitory effects of photoactivated OR141.

**Light source**	**A431**		**SCC7**		**B16–F10**	
	**EC50 (μM)**	**95% CI**	**EC50 (μM)**	**95% CI**	**EC50 (μM)**	**95% CI**
None (dark)	>100	–	>100	–	>100	–
White light LED	0.63	0.41–0.96	0.75	0.58–0.96	1.07	0.52–2.20
Sunlight	0.95	0.72–1.24	EC50: half maximal effective concentration			
Halogen	4.55	2.62–7.87	95% CI: 95% confidence interval			

### LED-activated OR141 induces cytotoxic effects in 3D tumor spheroids

**Figure 2:**
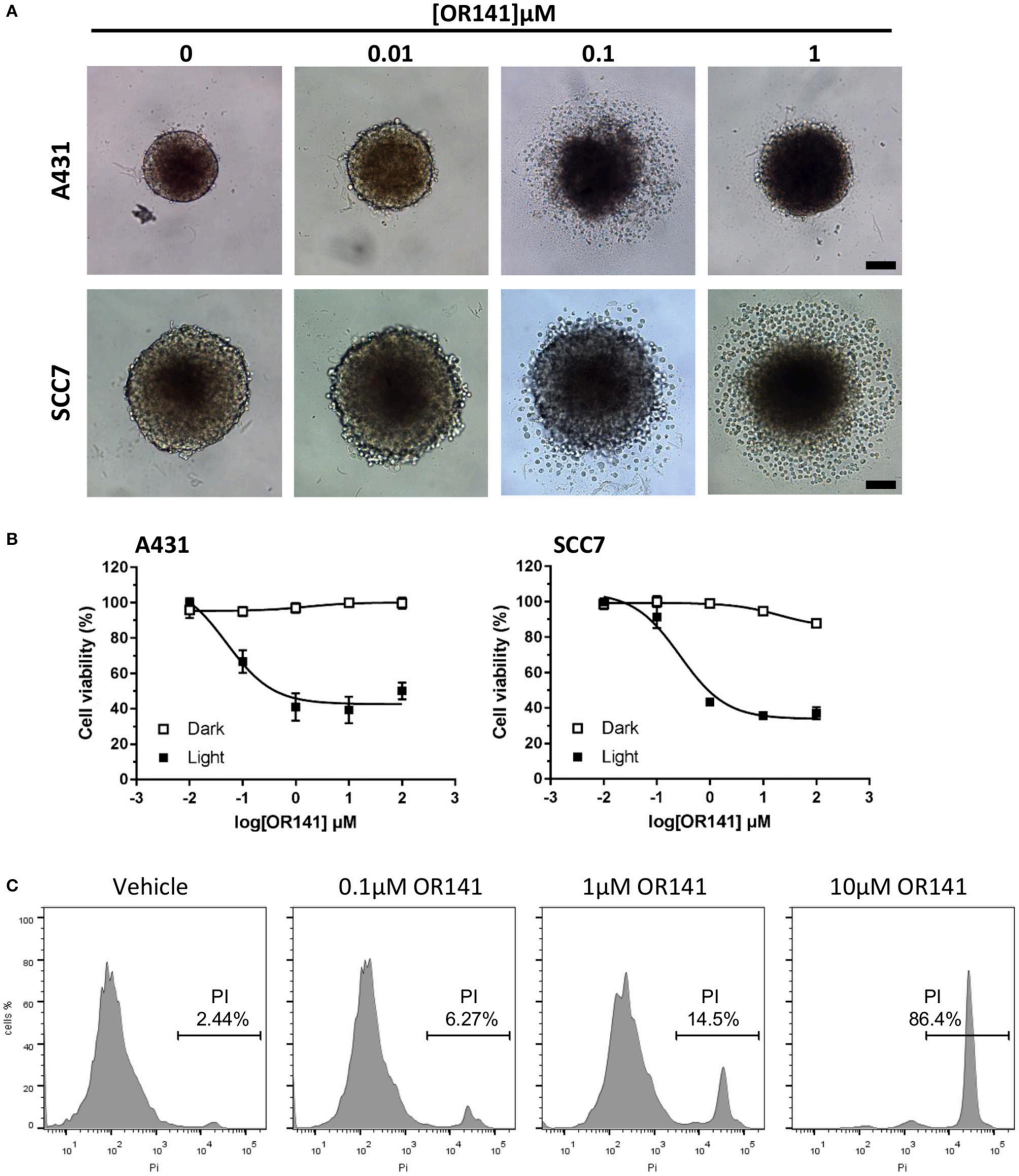
Cytotoxicity of OR141 in 3D tumor spheroids. Spheroids obtained from 3D cultures of A431 and SCC7 cancer cells were incubated with the indicated concentrations of OR141 for 4 h and further exposed to LED for 90 min (or maintained in the dark). **(A)** Representative pictures of 3D spheroids treated as indicated; scale bar = 100 μm; note that the dynamics of cell death in A431 spheroids was such that dead cells did not detach upon exposure to 1 μM OR141. **(B)** Dose-dependent effects of OR141 on the viability of A431 (left) and SCC7 (right) cells from treated spheroids (*n* = 6 spheroids per condition); data are normalized *vs*. values obtained with corresponding untreated spheroids. **(C)** Flow cytometry analysis of propidium iodide (PI)-positive SCC7 cells isolated from treated spheroids (*n* = 10 spheroids per condition); this experiment was repeated twice with similar results.

Since an obvious theoretical limitation of the use of white light for PDT is the limited depth of excitation, we aimed to verify whether diffusion of OR141 and penetration of LED light in 3D spheroids could lead to cytotoxic effects as observed in conventional 2D cancer cell cultures. In this study, we used ultra-low-attachment plates to generate 3D spheroids from A431 and SCC7 cancer cells (B16 cells did not give rise to spheroids). We observed a clear growth inhibition of OR141-treated spheroids in the presence of light as revealed by microscopy (Figure 2A) and measurements with Presto Blue cell viability reagent (Figure 2B) (as determined 24 h after 90 min LED light activation). To more directly identify tumor cell death, we also determined by flow cytometry the extent of iodide propidium (PI)-positive cancer cells isolated from treated SCC7 spheroids following trypsin digestion. A dose-dependent increase in the extent of PI-positive cancer cells was observed after exposure to LED-activated OR141 (Figure 2C).

### OR141 easily penetrates within the spheroid and LED-activation induces cell death in the spheroid depth

**Figure 3:**
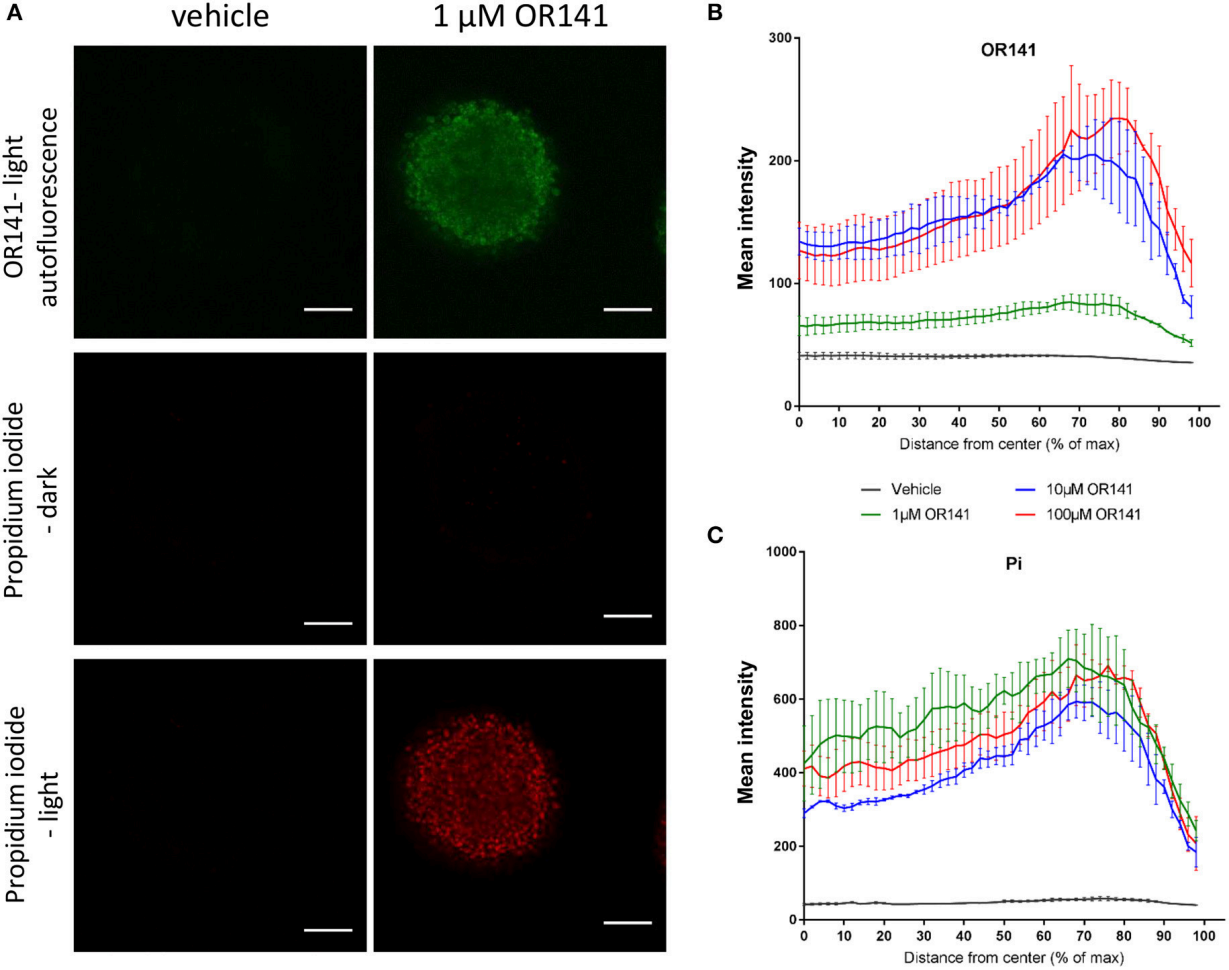
Diffusion of OR141 leads to tumor cell death in the depth of spheroids. SCC7 squamous cell carcinoma spheroids were incubated with the indicated concentrations of OR141 for 4 h and further exposed to LED for 90 min (or maintained in the dark). **(A)** Representative immunofluorescence pictures (confocal microscopy) of 3D spheroids revealing the distribution of OR141 (green autofluorescence) and propidium iodide (PI)-positive cells (red); scale bar = 100 μm. Quantification of **(B)** OR141 and **(C)** PI signals from the center to the periphery of spheroids (*n* = 3 spheroids per condition). Note that the decline of fluorescence signals at the farthest distance from the center is explained by the non-perfectly spheric form of the spheroids.

Although the above set of data confirmed the capacity of OR141 to exert cytotoxic effects within several layers of cancer cells, the core of the spheroid could not be sufficiently digested (to isolate cells for flow cytometry) and may have led to overestimate the amount of cell death. In a next series of experiments, we therefore used confocal microscopy to better discriminate the periphery from the deeper cell layers of the spheroids. In these experiments, the autofluorescence of OR141 also allowed us to confirm that the photosensitizer rapidly reached the most profound layers of cancer cells within spheroids (Figures 3A,B for quantification). Detection of PI-positive cancer cells (determined 24 h after 90 min LED light activation) also revealed that the whole spheroid was labeled from the periphery to the core (Figures 3A,B for quantification). It is worth noting that while the extent of OR141 staining was not maximal at 1 μM (see Figure 3B), this low concentration of OR141 led to a similar extent of cell death as obtained with higher OR141 concentrations (see Figure 3C).

### Optimization of OR141 formulation for *in vivo* use

**Figure 4:**
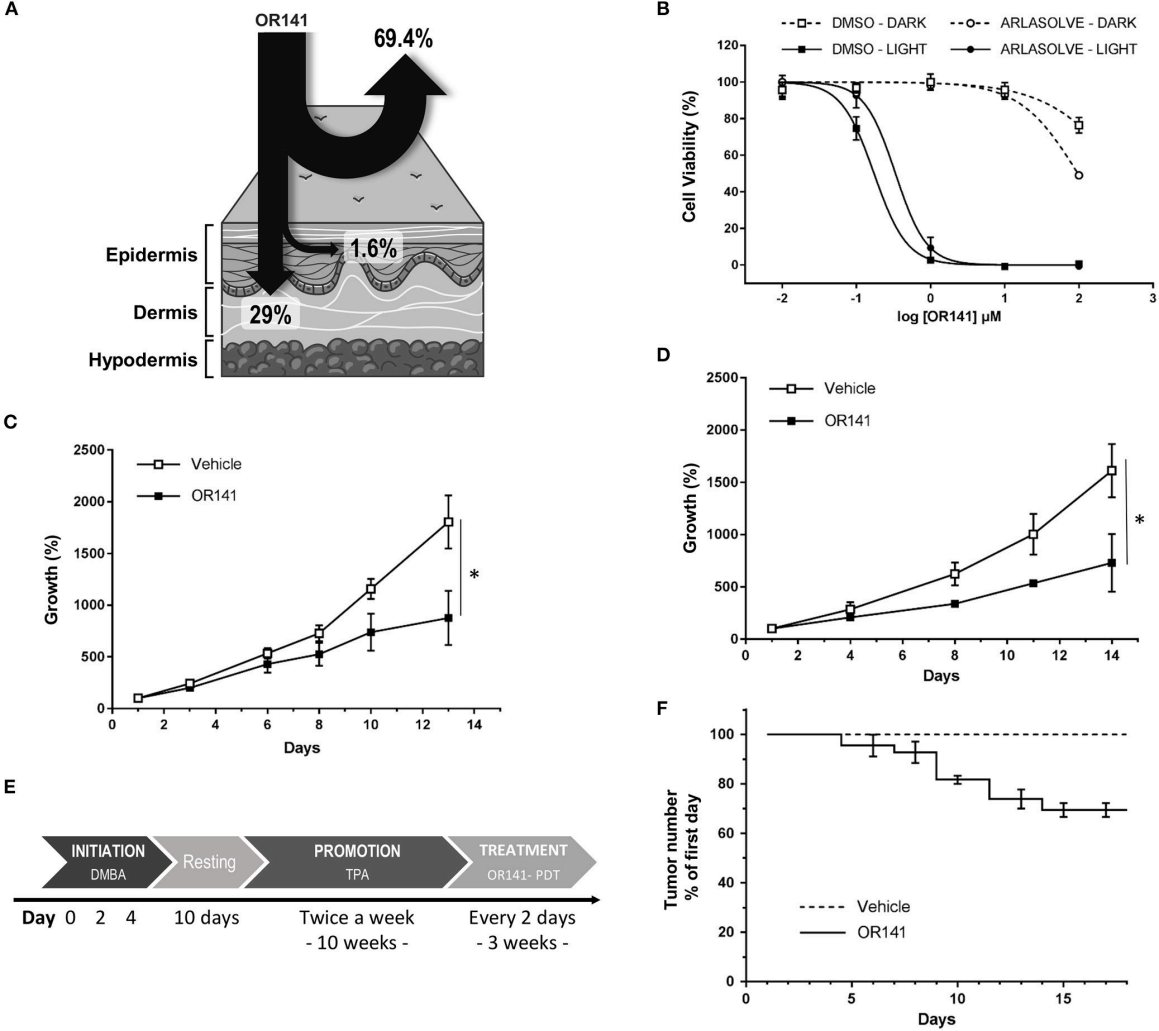
Inhibitory effects of OR141 formulated in a clinically compatible solvent on skin tumor growth and precancerous lesions in mice. **(A)** Distribution of OR141 dissolved in Arlasolve as assessed by using Franz cells mounted with human skin. **(B)** Dose-dependent effects of OR141 -formulated in Arlasolve or DMSO- on the viability of A431 skin cancer cells (*n* = 3). Growth inhibitory effects of LED-activated OR141 on A431 tumors in nude NMRI mice treated **(C)** either every 3 days with intraperitoneal (i.p.) administration of the PS or **(D)** three time a week with topical application of the PS dissolved in Arlasolve (*n* = 5 mice per group). **(E)** Two-step chemical induction protocol of mouse skin lesions including initiation of cellular damages with DMBA followed by promotion with TPA. **(F)** Effects of topically administered LED-activated OR141 on the number of skin precancerous lesions resulting from DMBA/TPA treatments (*n* = 5 mice), *P < 0.05.

OR141 is a rather lipophilic molecule with little solubility in aqueous solvent. In order to obtain a clinically compatible formulation and optimize bioavailability of OR141, we next evaluated several solvents. Maximal solubility was achieved with Arlasolve which permits to reach a maximum concentration of 14.1 mg/ml (Table 2). Skin penetration of OR141 dissolved in Arlasolve was then assessed *ex vivo* with Franz cells mounted with human skin. Of the applied dose on the external surface of the skin, 1.6 ± 0.3% was absorbed in the epidermis while 29 ± 15.5% of the compound reached the dermis (Figure 4A). Importantly, *in vitro* experiments confirmed that cytotoxic effects of OR141 formulated in Arlasolve were comparable to what was achieved with DMSO (the solvent used in previous experiments; Figure 4B). In the absence of light, a lack of OR141 activity was observed with Arlasolve and DMSO except at the highest concentration where despite darkness in the cell culture room, residual light may have induced OR141 photoactivation.

**Table 2:** Maximal OR141 solubility in various solvents.

**Solvents**	**OR141 max solubility** **(mg/ml)**
Arlasolve™ (Dimethyl Isosorbide)	14.1
Labrasol® (Caprylocaproyl polyoxyl-8 glycerides)	13.2
Transcutol® (Diethylene Glycol Monoethyl Ether)	13.0
PEG 400 (polyethylene glycol 400)	10.9
Labrafil® M1944 (Oleoyl polyoxyl-6 glycerides)	6.5
Ethanol	5.6
Plurol® Oleique (Polyglyceryl-3 Dioleate)	4.3
Labrafac™ lipophile WL 1349 (Triglycerides medium chain)	2.3
Isopropyl myristate	0.8
Oleic acid	0.6

### LED-activated OR141 inhibits the burden of tumors and pre-cancerous lesions in mice

For *in vivo* experiments, we used human A431 xenografts obtained after subcutaneous (s.c.) injection of 1 ^*^ 10^6^ cells in nude NMRI mice. When tumors reached a volume of 20 mm3, treatments were initiated. To evaluate possible differences between systemic delivery and topical application of OR141, we administered OR141 either intraperitoneally (40 mg/kg, twice a week) or by direct skin application (using a 20 mM solution, three times a week). Note that for mice treated with i.p. OR141 administration, local tumor illumination was performed to restrict photoactivation in the zone of the lesions whereas total body illumination was used for mice topically exposed to OR141 since photoactivation was *de facto* limited to the treated skin area. Non-treated groups were i.p. injected or topically treated with the vehicle and further exposed to the adequate light source. Both the systemic and topical routes led to significant tumor growth inhibitory effects as depicted in Figures 4C,D. We also chose to evaluate OR141 in a model of precancerous skin lesions induced by chemicals treatments over a period of 10 weeks. Exposure of mouse skin to DMBA and TPA (see detailed protocol in Figure 4E) led to the development of papillomas that eventually progress toward skin carcinoma cancers by invading underneath tissues. Here, we treated precancerous lesions by topical application of OR141 (dissolved in Arlasolve) and whole illumination of the mouse back. A significant decrease in the extent of papilloma burden was observed in mice exposed to PDT (vs. vehicle-exposed control mice) (Figure 4F). After 2 weeks of treatment with 3 rounds of PDT a week, 30% of the papillomas of treated mice disappeared *vs*. none in the control group (Figure 4F). The remaining 70% of lesions were composed of bigger papillomas that could not be eradicated with PDT alone.

## Discussion

Sources of conventional PDT often use red light to maximize depth of tissue penetration taking advantage of a small absorption peak of porphyrin-type PS around 635 nm. Blue light activation of porphyrins has however also been used exploiting the Soret band around 410 nm (25). Photoactivation in the blue wavelength region of the visible spectrum was shown to deliver more energy with a reduced irradiance and to be associated with lesser adverse effects for the patients (26). In this context, the absorption spectrum of OR141, the lead compound of a new non-porphyrin PS family, positions this drug as an attractive PDT modality. The absorption profile of OR141 actually fits LED light spectrum with a major peak in blue light (see Figures 1A,B). In this study, we expanded on the potential of OR141 by documenting that LED activation of this PS leads to significant *in vitro* and *in vivo* cytotoxic effects limiting tumor growth and preventing the burden of precancerous lesions in mice.

Although the observed growth inhibitory effects in mouse models *per se* confirms the therapeutic potential of this new PS, we used the more tractable 3D culture models to illustrate that both light and OR141 can penetrate deep enough to give rise to extensive cytotoxic effects. Taking advantage of OR141 autofluorescence we could indeed document its rapid diffusion and -upon LED photoactivation- its capacity to induce cell death in 400–600 μm diameter spheroids; the lack of illumination did not reveal any cytotoxic effects further proving the safety of this PS when not activated. It is also worth to emphasize that in mouse experiments, OR141 was i.p. injected (40 mg/kg, i.e., 1.2 mg OR141 per mouse) leading to a circulating concentration that we previously measured to be around 5 μg/ml (or 10 μM) after 6 h. This concentration is thus in the range of that used in spheroids experiments and confirms the feasibility of a systemic treatment. Data obtained using Franz's cell chambers documenting ~30% dermal OR141 absorption also support the potential of this treatment when topically administered, as confirmed by the growth inhibitory effects of photoactivated OR141 on skin (pre-)cancerous lesions (Figures 4D–F).

With the identification of Arlasove as a solvent compatible with human administration and skin absorption, this study supports further clinical development of OR141 as a PS that may offer several advantages besides previously identified specificities [i.e., lack of off-targets in the absence of light and ^1^O_2_ production in minimal pO_2_ environment (20)]. There is actually a need for white light-activated PS to facilitate patient care in small clinics or in dermatologists' offices. Moreover, beside a lower cost than that required for laser purchase and maintenance, a main advantage of LED light over lasers is a very broad light beam that allow larger areas to be treated in a shorter time slot; this may be particularly relevant for AK. When topically applied, it has also been suggested that PS could be directly activated by sunlight, a lower dose of PS being then coupled to a longer photoactivation period. Such modalities would diminish burning sensation which is critical since PDT-associated pain is known to reduce the efficacy of the procedure due to frequent early termination of treatments.

In conclusion, this study provides several lines of evidence supporting the clinical feasibility of LED light photoactivation of the non-porphyrin PS OR141 to treat skin (pre-) cancerous lesions. Although the extent of cell layers reached by LED lamp emission will remain lesser than with NIR-emitting lasers, we have demonstrated the capacity of OR141 exposed to white light LED to exert direct cytotoxic effects in 3D spheroids and mouse models. Together with the anticipated reduction in the adverse effects, in particular pain, known to be associated with conventional PDT, LED-activated PS, and in particular OR141, may be considered as a new component of the anticancer armamentarium for accessible tumors.

## Author contributions

BD, OR, and OF conceived the study, designed the experiments, wrote the manuscript and supervised the research. BD, EB, AP, CL, and AR performed the experiments. All the authors contributed to the interpretation of the results and critically revised the article.

### Conflict of interest statement

The authors declare that the research was conducted in the absence of any commercial or financial relationships that could be construed as a potential conflict of interest.
